# Outcomes with the Adjustable Transobturator Male System (ATOMS) for the Treatment of Male Stress Urinary Incontinence After Prostate Surgery and the Impact of Previous Radiotherapy

**DOI:** 10.1016/j.euros.2024.02.016

**Published:** 2024-03-04

**Authors:** Ingunn Roth, Patrick Juliebø-Jones, Christian Arvei Moen, Christian Beisland, Karin M. Hjelle

**Affiliations:** a Department of Urology, Haukeland University Hospital, Bergen, Norway; b Department of Clinical Medicine, University of Bergen, Bergen, Norway

**Keywords:** Incontinence, Adjustable transobturator male system continence device, Efficacy, Male stress urinary incontinence, Radiotherapy

## Abstract

**Background and objective:**

The adjustable transobturator male system (ATOMS) is an established treatment for patients with urinary incontinence after prostate surgery. Our objective was to evaluate the efficacy and the complication burdens associated with ATOMS with a focus on exploring the potential impact on previous radiotherapy (RT).

**Methods:**

We performed a retrospective analysis for consecutive patients who underwent ATMOS implantation procedure at a tertiary center over an 11-yr study period. Outcomes of interest were dryness at 3-mo follow up, postoperative complications (≤30 d), and late treatment failures (>30 d).

**Key findings and limitations:**

A total of 118 patients underwent ATOMS surgery performed by five different surgeons. Median follow-up was 67 mo (interquartile range 41–95). The mean 24-h pad count after surgery was 1.1 (range 0–8) and the mean reduction in pad weight was 179 g (range 0–1080). There was no significant difference in the reduction in pad use between groups with and without RT (−1.7 vs −2.4; *p* = 0.13). Multivariable analysis revealed that RT, degree of incontinence, and age were not risk factors for reoperation.

**Conclusions and clinical implications:**

ATOMS implantation is feasible in patients who have undergone prostate RT and patients with severe stress urinary incontinence after prostate surgery. We found that RT was not a risk factor for reoperation and there was no significant difference in pad weight reduction by RT status. This study offers new insight into potential incontinence surgery for male patients with stress urinary incontinence and previous RT.

**Patient summary:**

We assessed outcomes for patients who had an ATOMS (adjustable transobturator male system) device implanted to control stress urinary incontinence after prostate surgery. After implantation, 52.5% of the patients reported zero leakage and 39.9% reported only mild incontinence. Our results show that this device can improve continence after prostate surgery and is also suitable in patients who underwent radiotherapy.

## Take Home Message

This study offers new insight into a potential incontinence surgery option for male patients with stress urinary incontinence after prostate surgery and radiotherapy. Radiotherapy was not identified as a risk factor for reoperation.

## 1 Introduction

The psychological and physical sequelae of stress urinary incontinence (SUI) occurring after prostate surgery including radical prostatectomy (RP) impose a clinically significant burden on affected patients [1]. The impact on quality of life, including stress, limitations in activities of daily living, relationships, and sexual functioning cannot be overstated [2], [3]. Treatment options include conservative strategies such as pelvic-floor muscle training, lifestyle changes, and pharmacotherapy before evaluation for incontinence surgery is indicated [4]. Although rates of post-RP SUI have decreased in recent years because of improvements in surgical technique and a more comprehensive understanding of the underlying pathophysiology, it is reported that up to 40% of patients experience SUI beyond 12 mo, depending on the definition applied [5], [6]. In the setting of prostate cancer surgery, between 3% and 6% of patients will ultimately undergo further surgery as a formal treatment for SUI [7], [8], [9]. Risk factors in this setting include previous radiotherapy (RT), age, obesity, length of the membranous urethra, and surgeon experience, among others [10]. Before the advent of several alternative options, an artificial urinary sphincter (AUS) long served as the standard treatment, especially for moderate to severe incontinence, and dryness rates of up to 86% have been reported [11]. However, because of high costs, associated complications, and suboptimal reoperation rates, alternative interventions have been developed. These include sling systems such as the adjustable transobturator male system (ATOMS; Agency for Medical Innovations, Feldkirch, Austria) [12], which acts via noncircumferential urethral compression. However, despite its potential as an effective intervention, ATMOS outcomes remain under-reported, including the impact of previous RT. The importance of this research is arguably of ever-increasing relevance given the increasing volume of both RP and benign prostate surgeries being performed worldwide.

Our aim was to evaluate the efficacy and the complication burden associated with ATOMS implantation with a focus on exploring the potential impact of previous RT.

## 2 Patients and methods

We performed a retrospective analysis of consecutive patients who underwent ATOMS surgery between June 2012 and 2022. The study was registered as a departmental clinical audit and was therefore exempt from a requirement for ethical approval in accordance with local regulations. The indication for surgery was persistent postoperative SUI of at least 12 mo in duration after the primary intervention. All patients had a symptom burden refractory to conservative treatments that included lifestyle modifications and pelvic floor exercises. Urethral stricture was a contraindication to surgery. Patients with nocturnal urinary incontinence were instead considered as potential candidates for implantation of a different device. All patients underwent the following preoperative investigations: 24-h pad count and weight (measured for three consecutive days), frequency-volume charts, uroflowmetry, cystoscopy, and urodynamic evaluation. SUI was graded a mild (1–2 pads/d), moderate (3–5 pads/d), or severe (>5 pads/d).

All patients had a follow-up clinical visit at 3 mo after ATMOS surgery. Patients brought completed charts for 24-h pad count and weight to their visit so that the clinician could document and evaluate their symptom burden. If the patient experienced leakage, cushion adjustments were made. The maximum limit for cushion volume adjustment is 25 ml according to the device manufacturer. A record was kept for each patient to chart the cumulative cushion volume. Patients were routinely discharged at 3 mo unless further clinical follow-up was deemed necessary according to the clinician’s discretion and the individual patient’s needs. The electronic health records from the referring hospitals were also checked to account for any patient encounters including possible cushion adjustments.

### 2.1 Outcomes of interest

The primary outcome of interest was dryness at 3-mo follow-up. Complete dryness was defined as zero pads used per 24 h.

Secondary outcomes of interest included postoperative complications (≤30 d) graded according to the Clavien-Dindo system and late treatment failures (>30 d).

Data were collected for demographic characteristics, American Society of Anesthesiologists score, timing and type of previous prostate surgery, prior RT status, operation time, postoperative catheterization time, and length of hospital stay.

### 2.2 Statistical analysis

Logistic regression was performed to identify risk factors for reoperation. Implant patency curves were generated using the Kaplan-Meier method. A Wilcoxon rank-sum test was used to compare pad burden according to RT status. Fischer’s exact test was applied to compare preoperative urodynamic findings between the RT and no-RT groups. The reduction in the number of pads was compared between the RT and no-RT groups by calculating the difference before and after surgery (Δ) and then performing a an independent-sample t test. Analyses were performed using R v4.1.1 [13]. Statistical significance was set at *p* < 0.05.

### 2.3 Surgical technique

The surgical technique followed a standardized approach. Patients were positioned in the lithotomy position with flexion and abduction of the hips. A 14Fr urethral catheter was inserted before starting the surgery. Via perineal incision, the central cushion was placed against the bulbospongiosus muscle and the proximal bulbar urethra, and was then fixed with two mesh arms around the obturator foramina using a curved tunneler. After the air was removed, the cushion was filled with 10 ml of saline solution and allowed to equilibrate to atmospheric pressure, in accordance with the user manual. At this point of equilibrium, 1–2 ml of saline solution was added. Inguinal port placement was used for the first-generation ATOMS device (28 patients) and a dartos scrotal pouch was created to accommodate the port for second- and third generation ATOMS devices (90 patients). The operation was carried out under general anesthesia with antibiotic prophylaxis using intravenous aminoglycoside and a first-generation cephalosporin administered at induction.

## 3 Results

### 3.1 Patient characteristics

Over the 11-yr study period, 118 patients underwent ATOMS surgery performed by five different primary surgeons. All patients attended their 3-mo follow-up appointment and there was no loss to follow-up. Median follow-up was 67 mo (interquartile range [IQR] 41–95). Previous prostate surgery included RP (robot-assisted *n* = 98, open *n* = 14) and benign prostate surgery (transurethral resection of the prostate, TURP; *n* = 6). Of the 112 patients with previous treatment for prostate cancer, 39 (35%) had undergone RT (Table 1). None of the patients had undergone previous surgery for SUI. Median age was 64 yr (IQR 60–68) at the time of the original prostate surgery and 67 yr (IQR 65–70) at the time of ATMOS implantation.

**Table 1:** Baseline characteristics of the 118 patients

Parameter	Result
Mean age at prostate surgery, yr (range)	64 (60–68)
Mean age at ATOMS surgery, yr (range)	67 (65–70)
Median ASA score (IQR)	2 (2–2)
Median body mass index, kg/m^2^ (IQR)	26.5 (24.9–28.5)
Previous surgery, *n* (%)	
Robot-assisted laparoscopic RP	98 (83)
Open retropubic RP	14 (12)
Transurethral resection of the prostate	6 (5)
Prior radiotherapy, *n* (%)	39 (35)
Severity of preoperative SUI, *n* (%)	
Mild SUI	37 (31)
Moderate SUI	66 (56)
Severe SUI	15 (13)

IQR = interquartile range; ASA = American Society of Anesthesiologists; RP = radical prostatectomy; SUI = stress urinary incontinence.

### 3.2 Preoperative findings

Urodynamic studies revealed normal activity in 96 patients (81%), mild overactivity in 15 patients (13%), and reduced compliance in seven patients (6%). There was no difference in findings between the RT and no-RT groups (*p* = 0.4; Supplementary Table 1). The median postvoid residual volume was 0 ml (IQR 0–7). Preoperative SUI was mild in 31%, moderate, in 56%, and severe in 13% of the patients (Table 1). While there was no difference in the median number of preoperative pads between the no-RT and RT groups (3 vs 3; *p* = 0.1), the median preoperative weight was higher in the RT group (130 vs 201 g; *p* = 0.017).

### 3.3 Operative data

The median operative time was 39 min (IQR 33–45) and no intraoperative complications were recorded (Table 2). All procedures were successfully completed. The majority of the patients (86%) stayed in hospital for one night only.

**Table 2:** Summary of results

Parameter	Result
Perioperative data	
Median operating time, min (IQR)	39 (33–45)
Median hospital stay, d (IQR)	1 (1–1)
Mean catheterization time, d (range)	1 (1–7)
Degree of SUI at 3-mo follow-up, *n* (%)	
Complete dryness (0 pads/24 h)	62 (52.5)
Mild SUI	47 (39.9)
Moderate SUI	7 (5.9)
Severe SUI	2 (1.7)
Mean number of cushion adjustments (range)	2 (0–9)

IQR = interquartile range; SUI = stress urinary incontinence.

### 3.4 Follow-up

The median postoperative cushion volume was 7 ml (IQR 6–7). On average, patients required two cushion adjustments during the whole follow-up period (Table 2), with 33 patients (28%) requiring zero adjustments. The mean 24-h pad count after surgery was 1.1 (range 0–8) and the mean reduction in pad weight was 179 g (range 0–1080). Some 52.5% of the patients reported zero leakage. The majority of the remaining patients (39.9%) reported mild SUI only (Table 2). Comparison by RT status revealed that the median number of pads used (2 vs 1; *p* = 0.002) and median pad weight (11 vs 0 g; *p* = 0.013) were higher in the RT group than in the no-RT group (Table 3). However, there was no significant difference in the reduction in the number of pads used according to RT status (−1.7 vs −2.4; *p* = 0.13). Some 52 patients (44%) still required cushion adjustments at 12 mo after surgery. Preoperative and postoperative results according to previous surgery type are presented in Supplementary Table 2.

**Table 3:** Change in pad burden by radiotherapy status

Parameter	Median result (interquartile range)			*p* value a
	Overall (*n* = 118)	No radiation (*n* = 79)	Radiation (*n* = 39)	
Pad count preoperatively (*n*)	3 (2–4)	3 (2–4)	3 (3–4)	0.082
Pad weight preoperatively (g)	154 (68–266)	130 (59–238)	194 (101–374)	0.035
Data missing	1	1	0	
Pad count postoperatively (*n*)	1 (0–2)	1 (0–1)	2 (0.5–2)	0.002
Data missing	1	1	0	
Pad weight postoperatively (g)	0 (0–37)	0 (0–15)	10 (0–100)	0.037
Data missing	4	2	2	

a Wilcoxon rank-sum test.

### 3.5 Postoperative complications

The early complication rate was 2.5% (Table 4). All complications were minor (Clavien-Dindo grade <III). One patient was readmitted for pain, one for dyspnea, and two for lower urinary tract infection (Table 3).

**Table 4:** Summary of complications and late treatment failures

Parameter	Patients, *n* (%)
Intraoperative complications	0
Postoperative complications (≤30 d)	
Overall	3 (2.5)
Pain (CD grade I)	1 (0.8)
Dyspnea (CD grade I)	1 (0.8)
Urinary tract infection (CD grade II)	1 (0.8)
Treatment failures (>30 d)	
Total number of reoperations	19 (16)
Removal, replaced with AUS	7 (5.9)
Removal because of infection	1 (0.8)
Removal because of pain	2 (1.7)
Removal of port only because of erosion	2 (1.7)
Replaced port only because of persistent pain	7 (5.9)

CD = Clavien-Dindo; AUS = artificial urinary sphincter.

#### 3.5.1 Late treatment failures

Overall, 19 patients (16%) underwent reoperation for a device-related problem. One-third of these patients had undergone RT after RP. The rate of complete device removal was 8.5% (*n* = 10). Reasons for removal of the device included infection (*n* = 1), chronic pain (*n* = 2), and persistent leakage (*n* = 7). All patients with persistent leakage underwent complete device removal and AUS implantation in the same session. In two patients the port was removed for skin erosion. Seven patients needed port replacement because of discomfort and pain related to port dislocation (ex perineum or inguinal). Among the 19 reoperation cases, eight patients had mild, eight had moderate, and three had severe SUI.

In 12 of the cases (63%) requiring reoperation, this was performed within 12 mo of the ATOMS surgery. At 3.5 yr, the risk of implant removal plateaued (Supplementary Fig. 1). Multivariable analysis revealed that RT, degree of SUI, and age were not risk factors for reoperation (Table 5). When including both early complications and late treatment failures, the total complication rate was 18.6% (*n* = 22).

**Table 5:** Univariable and multivariable analysis of potential risk factors for reoperation

Variable	Patients	Univariable		Multivariable	
		OR (95% CI)	*p* value	OR (95% CI)	*p* value
Age at AMOS surgery	118	0.96 (0.90–1.04)	0.3	0.97 (0.90–1.04)	0.36
Stress urinary incontinence	118		0.4		0.44
Mild		Reference		Reference	
Moderate		0.65 (0.17–3.17)		0.68 (0.17–3.39)	
Severe		0.29 (0.03–2.00)		0.31 (0.04–2.17)	
Radiation therapy (yes vs no)	118	1.11 (0.36–3.15)	0.8	1.24 (0.39–3.63)	0.70

OR = odds ratio; CI = confidence interval.

## 4 Discussion

### 4.1 Key findings

Our results show a considerable reduction in the amount of leakage after ATMOS implantation. If reoperation occurred, this was most likely to be during the first 12 mo postoperatively. RT was not a risk factor for reoperation and there was no significant difference in the reduction in pad use by RT status. However, patients with previous RT had a significantly higher median pad weight both preoperatively and postoperatively. Surgeons should consider this during preoperative counseling in order to manage patient expectations.

The lack of higher reoperation risk and the lack of any significant difference in pad reduction between the groups in our study support the feasibility of ATOMS surgery in patients with a history of RT. It is recognized that deleterious effects associated with RT, such as decreased vascularity, tissue fibrosis, and poor healing, add to the surgical challenge [14]. However, by avoiding incision of the bulbospongiosus muscle and reducing the risk of erosion, ATMOS implantation offers an advantage over alternatives such as an AUS in the RT setting. It is anticipated that the demand for treatment options that are feasible for this complex patient group will increase given the rise in the absolute number of patients receiving adjuvant or salvage RT after RP [15]. This is especially the case given that fixed slings such as the AdVance XP device are considered to be contraindicated in this setting [16]. Another advantage of the ATOMS device is the ability to adjust the cushion volume postoperatively in the outpatient setting via the easily accessible scrotal port. By compressing the urethra noncircumferentially, patients can also void spontaneously. In comparison to AUS implantation, the ATMOS device requires a shorter operation time and is less prone to mechanical failure [17].

The question of whether RT negatively affects ATMOS outcomes, and, if so, to what extent, remains a matter of debate. In an Italian series recently described by Giammò and Ammirati [18], RT status did not affect continence outcomes. By contrast, Redmond et al [19] found that previous RT was associated with both failure to achieve continence and device removal in a Canadian series. It is likely that heterogeneity in study populations, differences in device generations, and variations in the continence definitions used contribute to these conflicting results.

ATOMS surgery is generally recommended for patients with mild or moderate SUI only. Similar to our series, other groups have expanded the indication to include severe SUI and demonstrated that the procedure is feasible for these cases [17]. However, as in the study by Mühlstädt et al [20], all of the results reveal that the extent of improvement is lower in severe SUI than in mild or moderate SUI.

Other potential determinants of ATMOS outcomes investigated include obesity, prior urethral surgery, and prior incontinence surgery [19]. Dorado and Angulo [21] recently developed a nomogram to predict ATOMS treatment success, but this is yet to be externally validated. Outcomes in our series are consistent with another large published series [17] and with a recent meta-analysis that revealed a dry rate of 69.3% and a global complication rate of 18.9% [22]; the device removal rate of 5.5% is also comparable to our result of 8.5%. Of note, removal rates as high as 20% have been reported [23]. Long-term studies are needed to assess patient satisfaction and possible decisional regret, including the population with previous RT. It is anticipated that findings from ongoing and prospective studies such as SATURN will help in understanding the true impact of RT [24].

### 4.2 Strengths and limitations

Our study has several limitations that should be acknowledged. These include the retrospective design, the single-center setting, the lack of a control group, and the heterogeneous study population, which included patients with previous RP or TURP. Of note, some of the study conclusions are based on a minority of patients who underwent RT (35%). It is also possible that a need for port replacement is attributable to surgeon error rather than RT. While the sample size can be considered quite large in the context of the literature, the study period for accumulation of this case volume was long. However, this is a consecutive patient series, with no patients lost to follow-up, that reflects real-world practice. Furthermore, median follow-up exceeded 5 yr and, to the best of our knowledge, this is the first series of its kind from Scandinavia. Practice patterns for reporting SUI severity vary in the literature between pad count and pad weight. Arguably, the latter is a more reliable tool given that, for example, pad size can vary. While we presented results in terms of pad count, we used pad weight when analyzing the impact of RT. Future prospective studies could consider reporting on the symptom burden once optimal cushion adjustment has been achieved.

## 5 Conclusions

ATOMS is an effective surgical option for the treatment of persistent SUI after prostate surgery. It has a low morbidity profile and the majority of patients achieve continence. ATOMS is a feasible option in patients who have undergone RT and patients with severe SUI. Our analysis showed that RT was not a risk factor for reoperation and there was no significant difference in pad weight reduction by RT status.

  ***Author contributions***: Patrick Juliebø-Jones had full access to all the data in the study and takes responsibility for the integrity of the data and the accuracy of the data analysis.

  *Study concept and design*: Juliebø-Jones, Roth, Hjelle, Beisland, Moen.

*Acquisition of data*: Juliebø-Jones, Roth, Hjelle.

*Analysis and interpretation of data*: Juliebø-Jones, Roth, Hjelle, Beisland, Moen.

*Drafting of the manuscript*: Roth, Juliebø-Jones.

*Critical revision of the manuscript for important intellectual content*: Juliebø-Jones, Roth, Hjelle, Beisland, Moen.

*Statistical analysis*: Moen, Roth.

*Obtaining funding*: None.

*Administrative, technical, or material support*: Hjelle, Beisland.

*Supervision*: Hjelle, Beisland.

*Other*: None.

  ***Financial disclosures:*** Patrick Juliebø-Jones certifies that all conflicts of interest, including specific financial interests and relationships and affiliations relevant to the subject matter or materials discussed in the manuscript (eg, employment/affiliation, grants or funding, consultancies, honoraria, stock ownership or options, expert testimony, royalties, or patents filed, received, or pending), are the following: None.

  ***Funding/Support and role of the sponsor*:** None.

  ***Data sharing statement*:** The data sets generated and/or analyzed during the study are available from the corresponding author on reasonable request.

  ***Ethics considerations*:** This study was conducted as a service evaluation and therefore ethical approval was not required in accordance with the local institutional practice.
