# Supplementary figures and images for: Outcomes with the Adjustable Transobturator Male System (ATOMS) for the Treatment of Male Stress Urinary Incontinence After Prostate Surgery and the Impact of Previous Radiotherapy
Source: Eur Urol Open Sci. 2024 Mar 4;62:68–73. doi: 10.1016/j.euros.2024.02.016 (PMC10925931; doi:10.1016/j.euros.2024.02.016)

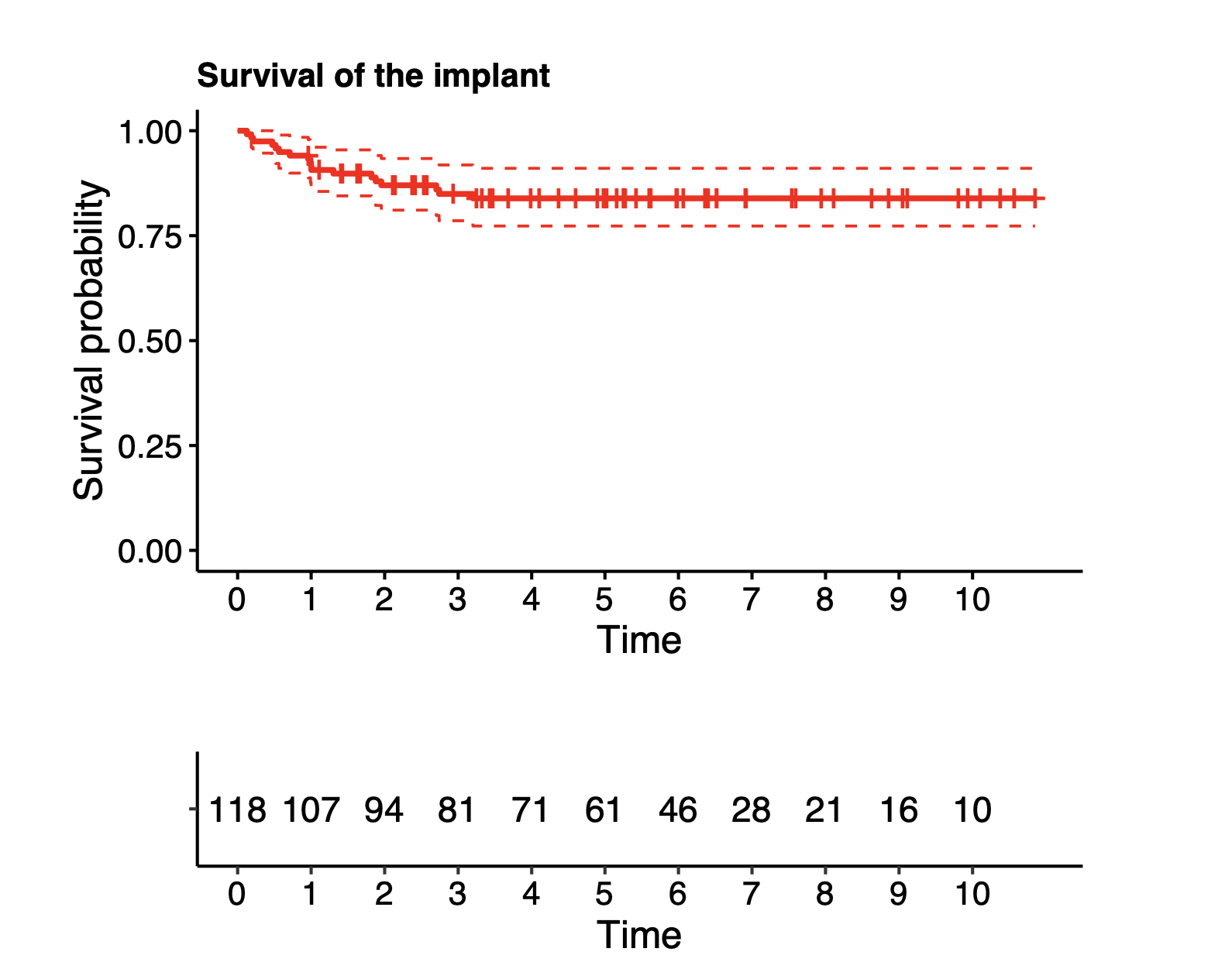


(Years)

(Years)

Supplementary Figure 1. Survival analysis of the implant

Supplement: Supplementary data 1 [file mmc1.docx]
