# Supplementary material for: Outcomes with the Adjustable Transobturator Male System (ATOMS) for the Treatment of Male Stress Urinary Incontinence After Prostate Surgery and the Impact of Previous Radiotherapy
Source: Eur Urol Open Sci. 2024 Mar 4;62:68–73. doi: 10.1016/j.euros.2024.02.016 (PMC10925931; doi:10.1016/j.euros.2024.02.016)
Supplement: Supplementary data 2 [file mmc2.docx]

| **Characteristic** | **Overall**, N = 118^1^ | **No radiation**, N = 79^1^ | **Radiation**, N = 39^1^ | **p-value**^2^ |
| --- | --- | --- | --- | --- |
| **Findings** |  |  |  | 0.4 |
| Non-obstructive, no overactivity | 96 (81%) | 67 (85%) | 29 (74%) |  |
| Non-obstructive, light overactivity | 10 (8.5%) | 6 (7.6%) | 4 (10%) |  |
| light overactivity, use Betmiga | 5 (4.2%) | 3 (3.8%) | 2 (5.1%) |  |
| Reduced compliance | 7 (5.9%) | 3 (3.8%) | 4 (10%) |  |
| ^1^n (%) | | | | |
| ^2^Fisher's exact test | | | | |

Supplementary Table 1. Comparison of pre-operative urodynamic findings according to previous radiotherapy status.
