# Supplementary material for: Outcomes with the Adjustable Transobturator Male System (ATOMS) for the Treatment of Male Stress Urinary Incontinence After Prostate Surgery and the Impact of Previous Radiotherapy
Source: Eur Urol Open Sci. 2024 Mar 4;62:68–73. doi: 10.1016/j.euros.2024.02.016 (PMC10925931; doi:10.1016/j.euros.2024.02.016)
Supplement: Supplementary data 3 [file mmc3.docx]

|  | **Pre-operative** | **Post-operative**  **(3 months)** |
| --- | --- | --- |
| **RALP (n=98)** |  |  |
| No SUI | - | 71 |
| Mild SUI | 35 | 15 |
| Moderate SUI | 52 | 4 |
| Severe SUI | 11 | 1 |
|  |  |  |
| **ORRP (n=14)** |  |  |
| No SUI | - | 3 |
| Mild SUI | 1 | 8 |
| Moderate SUI | 11 | 2 |
| Severe SUI | 2 | 1 |
|  |  |  |
| **TURP (n=6)** |  |  |
| No SUI | - | 4 |
| Mild SUI | 1 | 1 |
| Moderate SUI | 4 | 1 |
| Severe SUI | 1 | 0 |

RALP = Robotic assisted laparoscopic radical prostatectomy

ORRP = Open radical retropubic prostatectomy

TURP = transurethral resection of the prostate

SUI = Stress urinary incontinence

Supplementary table 2. Breakdown of results according to previous surgery type
